# The Deubiquitinase OTUD1 Influences HIV-1 Release by Regulating the Host Restriction Factor BST-2

**DOI:** 10.3390/v17020260

**Published:** 2025-02-14

**Authors:** Man-Di Zhang, Fan Chen, Wen-Qiang He, Ying Lu, Feng-Liang Liu, Hong-Guang Zhang, Liu-Meng Yang, Chun-Sheng Dong, Si-Dong Xiong, Yong-Tang Zheng

**Affiliations:** 1 State Key Laboratory of Genetic Evolution & Animal Models, Key Laboratory of Bioactive Peptides of Yunnan Province, KIZ-CUHK Joint Laboratory of Bioresources and Molecular Research in Common Diseases, Center for Biosafety Mega-Science, Kunming Institute of Zoology, Chinese Academy of Sciences, Kunming 650223, China; 18761931560@139.com (M.-D.Z.); luying92@foxmail.com (Y.L.);; 2 KIZ-SU Joint Laboratory of Animal Model and Drug Development, College of Pharmaceutical Sciences, Soochow University, Suzhou 215021, China; 3 Jiangsu Key Laboratory of Infection and Immunity, Institutes of Biology and Medical Sciences, Soochow University, Suzhou 215021, China; 4 University of Chinese Academy of Sciences, Beijing 100049, China

**Keywords:** deubiquitinase, OTUD1, BST-2, HIV-1

## Abstract

Bone marrow stromal cell antigen 2 (BST-2) is a restriction factor for human immunodeficiency virus type I (HIV-1) and plays an important role in regulating the release of viral particles. However, the antiviral efficacy of BST-2 is antagonized by the HIV-1-encoded accessory protein Vpu, which facilitates the degradation of BST-2 by recruiting E3 ubiquitin ligase β-TrCP. The involvement of deubiquitinases (DUBs) in counteracting BST-2 ubiquitination and influencing its stability during HIV-1 infection remains inadequately explored. In this study, we conducted a small interfering RNA (siRNA) screening of human DUBs and determined that OTUD1 interacts with BST-2, leading to a reduction in its K48- and K63-linked ubiquitination. This reduction increases BST-2 protein stability, and subsequently inhibits HIV-1 release. Our findings reveal a novel regulatory mechanism by which DUBs influence the stability of the HIV-1 restriction factor BST-2 to dampen viral release, providing a potential therapeutic target for HIV-1 antiviral intervention.

## 1. Introduction

Human cells evolve various antiviral proteins, known as host restriction factors, to resist human immunodeficiency virus type I (HIV-1) infection through the process of host–virus co-evolution. These restriction factors can exert antiviral effects at multiple stages of the viral life cycle, including viral entry, reverse transcription, integration, viral protein translation, and virion packaging and release, constituting the first barrier of the intrinsic cellular response to infection [1]. Multiple host restriction factors have been identified to date. For example, the apolipoprotein B mRNA-editing catalytic polypeptide like 3 (APOBEC3) family, which inhibits viral replication by introducing base mutations during reverse transcription [2]; the α-isoform of tripartite motif-containing protein 5 (TRIM5α), which causes premature uncoating and disassociation of the capsid core [3,4]; and bone marrow stromal cell antigen 2 (BST-2), which inhibits viral particle release by tethering viruses to the cell membrane [5,6].

BST-2, also known as tetherin, CD317, or HM1.24, is an interferon-induced type II transmembrane glycoprotein. Human BST-2 comprises 180 amino acids and exhibits a molecular weight of 29–33 kDa due to protein glycosylation [7]. Its antiviral potential was first identified in HIV-1 in 2008, and has since been observed in other enveloped viruses, including hepatitis B virus (HBV), hepatitis C virus (HCV), simian immunodeficiency virus (SIV), and Herpes simplex virus (HSV) [5]. This antiviral function is attributed to the unique topology of BST-2, which includes an N-terminal cytoplasmic tail, a transmembrane helix, a coiled coil ectodomain, and a C-terminal glycosylphosphatidylinositol (GPI) anchor [8]. The most widely accepted model posits that BST-2 forms parallel dimers through its transmembrane domain, anchoring to the host cell membrane, while the GPI anchor inserts into the viral envelope. This configuration tethers virions to the membrane surface of infected cells, preventing their release. The surface-captured virions are subsequently internalized and degraded through the endosomal/lysosomal pathway [9]. Beyond its antiviral impacts, BST-2 is also implicated in cancer metastasis, regulating T cell responses and autophagy [10].

The ubiquitin–proteasome system (UPS) is a primary protein degradation system in eukaryotic cells, crucial for regulating various cellular processes. The system consists of ubiquitin (Ub), ubiquitin-activating enzyme E1, ubiquitin-conjugating enzyme E2, ubiquitin ligase E3, deubiquitinases (DUBs), and 26S proteasome. These are involved in a process in which the E1 ubiquitin-activating enzyme first binds to ubiquitin molecule in an ATP-dependent manner to form high-energy thiol ester bonds. The ubiquitin molecules are then transferred to the mercaptan group of the E2 ubiquitin binding enzyme; finally, ubiquitin molecules are recognized by the E3 ubiquitin ligase and transferred to lysine residues of the target protein to form an isopeptidase bond, thus enabling the ubiquitization modification process [11]. HIV-1 has developed strategies to exploit the UPS to encode accessory proteins to antagonize host restriction. For example, the restriction factor APOBEC3G is targeted by the viral protein Vif, which induces its ubiquitination and degradation via the UPS [12]. Similarly, the antiviral function of BST-2 is antagonized by the accessory protein Vpu, which recruits the E3 ubiquitin ligase adapter β-TrCP, leading to the ubiquitination of BST-2 at phosphorylated serine residues S52 and S56 within the DSGXXS motif. This ubiquitination directs BST-2 toward degradation through either the targeted proteasome or lysosome, thereby reducing its capacity to inhibit viral release and weakening its antiviral response [13,14,15].

DUBs play a critical role in reversing the ubiquitination process, thereby modulating the stability and function of various proteins. The human genome encodes approximately 100 DUBs, many of which have been implicated in the regulation of viral replication through targeted deubiquitination [16]. Previous studies have shown that USP15 degrades HIV-1 Nef and Gag proteins, subsequently inhibiting HIV-1 replication [17]. In contrast, the DUB USP7 enhances HIV-1 production by stabilizing HIV-1 Tat protein [18], while USP21 inhibits viral replication by deubiquitinating Tat or down-regulating cyclin T1 expression [19]. Additionally, USP49 acts directly on APOBEC3G, reducing its ubiquitination and restoring its expression, thereby exerting an anti-HIV-1 effect [12]. To systematically investigate whether any DUBs are involved in regulating BST-2 ubiquitination and the subsequent release of HIV-1 virions, we conducted a genome-wide small interfering RNA (siRNA) screening of human DUBs. Our results identified OTUD1 as a key regulator of BST-2 membrane expression. Notably, OTUD1 interacts with BST-2, reducing its K48- and K63-linked ubiquitination at Lys21, thereby stabilizing its surface expression. More importantly, OTUD1 increases the inhibitory effect of BST-2 on HIV-1 release, highlighting its potential as a therapeutic target for anti-HIV-1 drug development.

## 2. Materials and Methods

Cell culture and transfection. HEK293T, HeLa, HeLa-CD4, and Jurkat cells were maintained in the laboratory and cultured in Dulbecco’s Modified Eagle Medium (DMEM, BasalMedia, L310KJ, Shanghai, China) or RPMI-1640 supplemented with 10% fetal bovine serum (Eallbio; 0.3U16001DC, Beijing, China), 100 U/mL penicillin, and 100 mg/mL streptomycin. The cells were incubated at 37 °C in a 5% CO_2_ environment. HIV-1(NL4-3) virality was maintained in our laboratory. Transient transfection was performed using Longtrans (UcallM Biotechnology, Wuxi, China) for DNA plasmid transfection and a riboFECT CP Transfection Kit (C10511-05, Guangzhou, China) for siRNA transfection.

Plasmids and reagents. A siRNA library of 98 human-derived DUB genes and OTUD1-siRNA (SIGS0001985-1, Guangzhou, China) was purchased from RiboBio (STLC001, Guangzhou, China). The plasmids pCMV3-BAP1-FLAG (HG18454-CF, Beijing, China) and pCMV3-OTUD1 (HG24641-UT, Beijing, China) were obtained from Sino Biological, while plasmids pCMV-USP26-FLAG (P22744, Shenzhen, China), pcDNA3.1-USP44-3×FLAG (P11718, Shenzhen, China), and pCMV-USP45-FLAG (P21517, Shenzheng, China) were purchased from Shenzhen Yibaishun Technology Co., Ltd. (Shenzhen, China). Ubiquitin-associated plasmids (Myc-Ub, HA-Ub-K6, HA-Ub-K11, HA-Ub-K27, HA-Ub-K29, HA-Ub-K33, HA-Ub-K48, and HA-Ub-K63) were generously donated by Professor Zheng Hui of Soochow University. The p(NL4-3), p(NL4-3)ΔVpu, and HA-BST-2 plasmids were preserved by this laboratory. OTUD1, BST-2, and their mutant plasmids were synthesized by inserting the corresponding gene fragments into the pCMV-HA, pCMV-FLAG, pCMV-Myc, and pcDNA3.1(+) vectors. The resulting plasmids were named FLAG-OTUD1, FLAG-BST-2, FLAG-OTUD1-C320S, FLAG-BST-2-K18R, FLAG-BST-2-K21R, FLAG-BST-2-K47R, FLAG-BST-2-K79R, FLAG-BST-2-K106R, FLAG-BST-2-K111R, FLAG-BST-2-K112R, FLAG-BST-2-K126R, FLAG-BST-2-K151R, and FLAG-BST-2-K152R. Site-directed mutant plasmid primers were designed using the Agilent Technologies primer design tool (https://www.agilent.com.cn/store/primerDesignProgram.jsp, accessed on 21 May 2020). The primer sequences are provided in Supplementary Table S1.

DUB siRNA library screening. Cells were seeded at a density of 1 × 10^5^ in 48-well plates, with each well being transfected with a different DUB-specific siRNA. After 48 h, the cells were collected and labeled with APC anti-human BST-2 antibody (BioLegend, 348402, San Diego, CA, USA, 1:1000) at room temperature for 20 min, and subsequently washed twice. The FACSVerse™ system (BD, Franklin Lakes, NJ, USA) was then used to detect the resuspended cells, and data were analyzed using FlowJo v10.6.2.

RNA isolation and real-time polymerase chain reaction (RT-qPCR). Total RNA was extracted from cells using TRIzol reagent (Yuan Ye, r21086, Shanghai, China). The cDNA was synthesized from 500 ng of total RNA using 5 × All-In-One RT Master Mixes (Trans, AE311-03, Beijing, China). mRNA levels were quantified by RT-qPCR using 2 × SYBR Green qPCR Master Mix (Vazyme; Q511-02, Nanjing, China). Primer sequences are listed in Supplementary Table S2. GAPDH was used as the reference gene to normalize OTUD1 and BST-2 mRNA expression in all samples. Relative expression was calculated using the 2^−ΔΔCt^ method [20].

**Figure 1:**
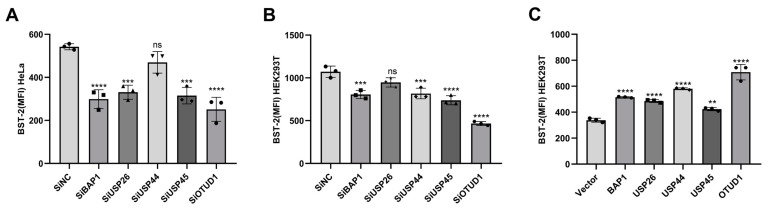
OTUD1 strongly regulates BST-2 membrane expression. (**A**) HeLa cells were transfected with BAP1, USP26, USP44, USP45, OTUD1, and five DUB-siRNAs, with the MFI of BST-2 on the membrane surface then being detected by flow cytometry after 48 h. (**B**) HEK293T cells, which lack endogenous BST-2, were co-transfected with BST-2 and BAP1, USP26, USP44, USP45, OTUD1, and five DUB-siRNAs, with the MFI of BST-2 on the membrane surface then being detected by flow cytometry after 48 h. (**C**) HEK293T cells were co-transfected with BST-2 and BAP1, USP26, USP44, USP45, and OTUD1, with the MFI of BST-2 on the membrane surface then detected after 48 h. *n* = 3, bars and error bars indicate mean ± SD. Paired *t*-tests were used to compare groups. *n* = 3. Values of ** *p* < 0.01, *** *p* < 0.001, and **** *p* < 0.0001 were considered statistically significant (NS, not significant).

PR-619 inhibition assay. HEK293T cells were transfected with (0.3 μg) BST-2-FLAG and (0.2 μg) Vpu-GFP for 32 h, and PR-619 (LifeSensors, SI9619, Malvern, PA, USA) of 15 μM, and 20 μM were added for 16 h, respectively. The treated HEK293T cells were centrifuged at 800 rpm, washed twice with pre-cooled PBS, and then labeled with phycoerythrin–propidium iodide (PE-PI, Abcam, ab14083, Cambridge, UK) and APC anti-human BST-2 (BioLegend, 348402, San Diego, CA, USA) antibodies at room temperature for 20 min. After washing twice, the FACSVerse™ system (BD) was then used to detect the resuspended cells, and data were analyzed using FlowJo v10.6.2. The gating strategy is depicted in Figure 1A.

Cycloheximide (CHX) chase assay. HEK293T cells were transfected with vectors expressing BST-2 and OTUD1 for 36 h. Subsequently, CHX (100 μg/mL) (MedChemExpress, 66-81-9, USA) was added at 0 h, 6 h, and 12 h post-treatment. After cell collection, Western blot analysis was performed to detect FLAG-BST-2 protein levels.

Western blotting. Proteins were extracted using RIPA buffer, then separated using sodium dodecyl sulfate-polyacrylamide gel electrophoresis (SDS-PAGE) and transferred onto polyvinylidene fluoride (PVDF) membranes (Merck Millipore, 2033565, Darmstadt, Germany). The membranes were blocked with 5% skim milk for 2 h at room temperature, followed by overnight incubation with specific primary antibodies, including OTUD1 (Abcam, ab122481, Cambridge, UK, 1:1000), BST-2 (Proteintech, 13560-1-APC, Chicago, IL, USA, 1:1000), FLAG (ABclonal, AE063, Wuhan, China, 1:2000), HA (ABclonal, AE008, Wuhan, China, 1:2000), and Myc (ABclonal, AE010, Wuhan, China, 1:2000). The membranes were then incubated for 1 h at room temperature with horseradish-peroxidase (HRP)-conjugated goat anti-mouse (ABclonal, AS003, Wuhan, China, 1:1000) and HRP-conjugated goat anti-rabbit secondary antibodies (ABclonal, AS014, Wuhan, China, 1:1000). Protein detection was performed using ECL luminescent reagent (NCM Biotech, Suzhou, P10200, China).

Co-immunoprecipitation. Cells were lysed using western and IP cell lysate buffer (Beyotime, P0013, Shanghai, China) with protease inhibitor phenylmethylsulfonyl fluoride (PMSF, 1:1000). The lysates were centrifuged at 12,000 rpm for 5 min, at 4 °C, and the supernatants were incubated overnight at 4 °C with corresponding immunomagnetic beads conjugated with anti-Flag (Selleck, B26101, Houston, TX, USA) or anti-HA (SelleckBimake, B26201, Houston, TX, USA). The beads were then washed 3–5 times with 0.05% 1× PBST before analysis by Western blot.

Statistical analysis. Flow cytometry data were analyzed using FlowJo v10 software. Other data were analyzed using Graph Pad Prism v8.0. Group comparisons were made using Student’s two-tailed *t*-test for statistical significance. Values of * *p* < 0.05, ** *p* < 0.01, *** *p* < 0.001, and **** *p* < 0.0001 were considered statistically significant (NS, not significant). All data are presented as mean ± standard deviation (SD).

## 3. Results

### 3.1. OTUD1 Significantly Enhances BST-2 Surface Expression

The human genome encodes approximately 100 putative DUBs, categorized into five distinct families: ubiquitin-specific proteases (USPs), ovarian tumor proteases (OTUs), ubiquitin carboxyl-terminal hydrolases (UCHs), Josephin domain proteins, and the JAB1/MPN/MOV34 (JAMM) metalloproteases family [21]. To investigate the potential role of DUBs in regulating BST-2 expression, we performed a comprehensive siRNA library screening on 98 individual DUBs in HeLa cells, which are known for their high endogenous expression of BST-2. A total of 48 h after the transfection of siRNA, flow cytometry was performed as the first round of detection for the mean fluorescence intensity (MFI) of the BST-2 surface, and we found that BST-2 showed varying degrees of decline after sixteen DUBs (ATXN3, TNFAIP3, USP7, BAP1, USP52, USP16, ATG4B, USP24, BRCC3, USP26, USP44, USP32, MPND, USP45, ATXN3L, and OTUD1) were knocked down (Figure S1). To avoid experimental errors as much as possible, we conducted a second round of screening with these sixteen DUBs. Five DUBs (BAP1, USP26, USP44, USP45, and OTUD1) were found to cause a decrease in BST-2 or a mean lower than negative control (Figure S2). For further verification, we performed a third round of screening with HeLa cells. After three rounds of repeated screening, we found that OTUD1 seemed to have the most significant effect on BST-2 (Figure 1A). Subsequently, further experiments on HEK293T cells expressing exogenous BST-2 demonstrated that silencing these five DUBs led to comparable reductions in BST-2 surface levels (Figure 1B). In contrast, overexpression of these DUBs enhanced BST-2 levels on the cell surface expression (Figure 1C). However, among the 98 DUBs analyzed, we found that OTUD1 seemed to have the most significant effect on BST-2. Therefore, OTUD1 was selected for the mechanism study.

### 3.2. OTUD1 Enhances BST-2 Protein Levels Through Direct Interaction

**Figure 2:**
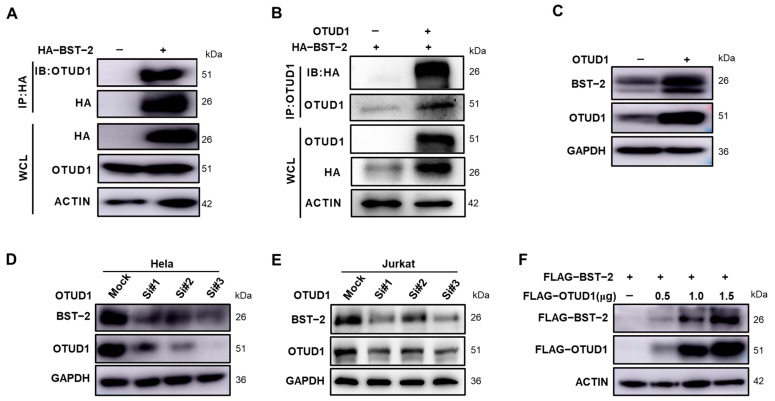
OTUD1 interacts with BST-2 and significantly enhances its protein levels. (**A**) HA-BST-2 plasmids were transfected into HEK293T cells, and proteins were collected after 48 h. HA-BST-2 was then incubated with HA immunomagnetic beads, followed by Western blot detection of OTUD1 protein. (**B**) HEK293T cells were co-transfected with OTUD1 and HA-BST-2 plasmids, and proteins were collected after 48 h. OTUD1 was then immunoprecipitated with OTUD1 antibody for 6 h, followed by the addition of protein A/G beads overnight. HA-BST-2 protein levels were subsequently detected by Western blot. (**C**) OTUD1 was overexpressed in HeLa cells, with protein levels of endogenous BST-2 assessed by Western blot after 48 h. (**D**) HeLa cells were transfected with OTUD1-specific siRNA, with protein levels of endogenous BST-2 detected by Western blot after 48 h. (**E**) Jurkat cells were transfected with OTUD1-specific siRNA, with protein levels of endogenous BST-2 detected by Western blot after 48 h. (**F**) HEK293T cells were transfected with 0.5 μg of FLAG-BST-2 and 0, 0.5 μg, 1.0 μg, or 1.5 μg of FLAG-OTUD1, with protein levels of FLAG detected by Western blot after 48 h. *n* = 3.

Given the substantial increase in BST-2 surface expression induced by OTUD1, their potential interaction was investigated. Co-immunoprecipitation showed that OTUD1 may interact with BST-2 protein (Figure 2A,B). To further explore whether OTUD1 influences BST-2 protein levels, experiments were conducted to assess its effect on BST-2 expression. Consistent with the screening results, overexpression of OTUD1 in HeLa cells led to an elevation in the total cellular levels of BST-2 (Figure 2C). Conversely, OTUD1 knockdown resulted in a reduction in BST-2 levels (Figure 2D). These effects were also observed in Jurkat cells (Figure 2E). The dose-dependent effect of BST-2 appears to have been determined in HEK293T cells (Figure 2F). Collectively, these findings indicate that OTUD1 not only interacts with BST-2, but also plays a crucial role in regulating BST-2 protein levels.

### 3.3. OTUD1 Enhances BST-2 Stability

**Figure 3:**
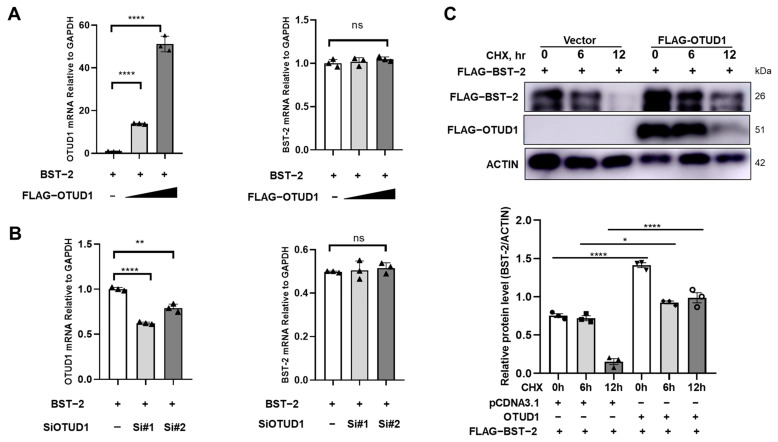
OTUD1 significantly enhances BST-2 protein stability. (**A**) OTUD1 was overexpressed in HeLa cells, and RT-qPCR was conducted to detect mRNA levels of OTUD1 and BST-2 at 48 h post-transfection. (**B**) HeLa cells were transfected with OTUD1-specific siRNA, and RT-qPCR was conducted to detect mRNA levels of OTUD1 and BST-2 at 48 h post-transfection. (**C**) After Flag-OTUD1 and Flag-BST-2 were co-transfected in HEK 293T cells. Cells were treated with CHX (100 μg/mL), and BST-2 protein levels were detected by Western blot at different time points (0, 6, 12 h) after treatment. Relative expression of mRNA was calculated using the 2^−ΔΔCt^ method. *n* = 3. Bars and error bars indicate mean ± SD. Paired *t*-tests were used to compare groups. *n* = 3. * *p* < 0.05, ** *p* < 0.01, **** *p* < 0.0001. ns, not significant.

Based on the above findings, the impact of OTUD1 on BST-2 expression was investigated at the transcriptional level. The qPCR results revealed that neither overexpression (Figure 3A) nor down-regulation (Figure 3B) of OTUD1 significantly affected BST-2 mRNA levels in HeLa cells, suggesting that OTUD1-mediated enhancement of BST-2 protein levels is not due to transcriptional regulation. However, when treated with the protein synthesis inhibitor CHX, OTUD1 significantly enhanced the stability of the BST-2 protein (Figure 3C), suggesting that the regulatory effects occur post-translationally.

### 3.4. OTUD1 Affects BST-2 Ubiquitination Levels

**Figure 4:**
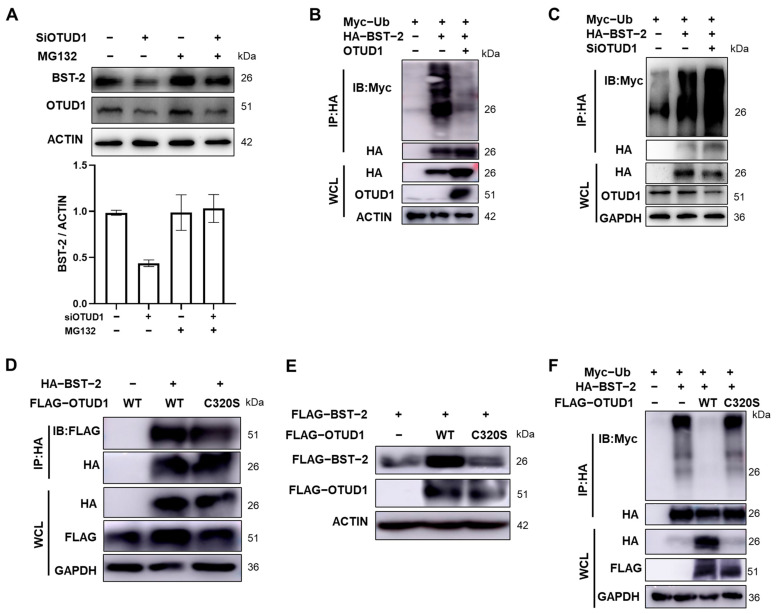
OTUD1 regulates BST-2 ubiquitination. (**A**) HeLa cells were transfected with OTUD1-specific siRNA for 36 h, followed by treatment with MG132 (10 μM) for 12 h. BST-2 protein levels were then detected by Western blot. (**B**) HEK293T cells were transfected with Myc-Ub, HA-BST-2 plasmids, and OTUD1, followed by co-incubation with HA immunomagnetic beads after 48 h. BST-2 ubiquitination levels were then detected by Western blot. (**C**) HEK293T cells were co-transfected with Myc-Ub, HA-BST-2 plasmids, and OTUD1-specific siRNA, followed by co-incubation with HA immunomagnetic beads after 48 h. BST-2 ubiquitination levels were then detected by Western blot. (**D**) HEK293T cells were transfected with HA-BST-2 plasmids, FLAG-OTUD1, or FLAG-OTUD1 C320S, followed by co-incubation with HA immunomagnetic beads after 48 h. FLAG protein was then detected by Western blot. (**E**) HEK293T cells were transfected with FLAG-BST-2, FLAG-OTUD1, or FLAG-OTUD1 C320S, with BST-2 protein levels detected by Western blot after 48 h. (**F**) HEK293T cells were transfected with Myc-Ub, HA-BST-2, FLAG-OTUD1, or FLAG-OTUD1 C320S, followed by co-incubation with HA immunomagnetic beads after 48 h. BST-2 ubiquitination levels were then detected by Western blot. *n* = 3.

Further investigation into the underlying mechanism revealed that OTUD1 influences BST-2 protein stability by modulating its ubiquitination. In order to investigate whether the effect of OTUD1 on BST-2 involves the ubiquitin proteasome pathway, we treated it with proteasome inhibitor MG132. Our results showed that treatment with the proteasome inhibitor MG132 mitigated the degradation of endogenous BST-2 mediated by OTUD1-siRNA in HeLa cells (Figure 4A). Next, the role of OTUD1 in regulating BST-2 ubiquitination was assessed. We found that OTUD1 overexpression in HEK293T cells led to a significant reduction in BST-2 ubiquitination (Figure 4B), while OTUD1 knockdown resulted in increased ubiquitination levels (Figure 4C). These findings suggest that OTUD1 stabilizes the BST-2 protein by targeting its ubiquitination. To further elucidate the role of OTUD1 activity, the cysteine residue at position 320, critical for its enzymatic function [22], was mutated to serine (OTUD1-C320S). While this mutation did not disrupt the interaction between OTUD1 and BST-2 (Figure 4D), it abolished the ability of OTUD1 to reduce BST-2 ubiquitination (Figure 4E,F). These results demonstrate that the effects of OTUD1 on BST-2 stability are dependent on its DUB activity.

### 3.5. OTUD1 Reduces K48 and K63-Linked Ubiquitination of BST-2

**Figure 5:**
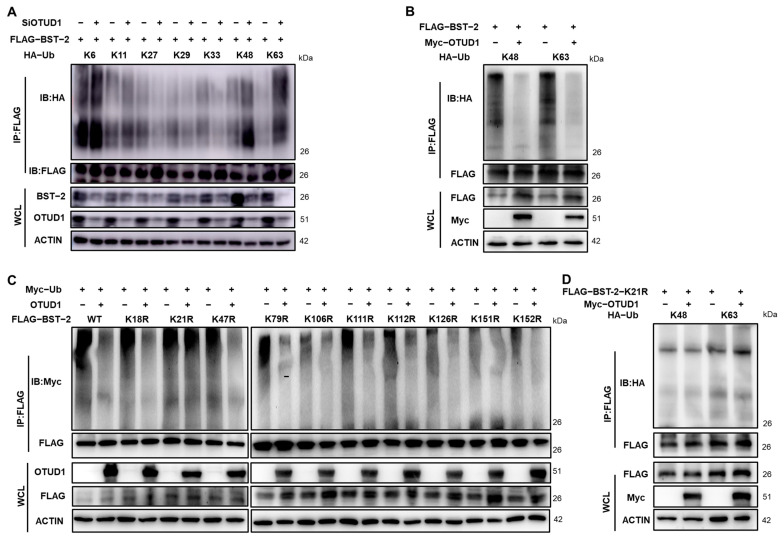
OTUD1 reduces K48- and K63-linked ubiquitination of BST-2 at lysine 21. (**A**) HEK293T cells were co-transfected with FLAG-BST-2, OTUD1-specific siRNA, HA-Ub-K6, HA-Ub-K11, HA-Ub-K27, HA-Ub-K29, HA-Ub-K33, HA-Ub-K48, and HA-Ub-K63, followed by co-incubation with FLAG immunomagnetic beads after 48 h. BST-2 ubiquitination levels were then detected by Western blot. (**B**) HEK293T cells were transfected with FLAG-BST-2, Myc-OTUD1, HA-Ub-K48, or HA-Ub-K63, followed by co-incubation with FLAG immunomagnetic beads after 48 h. BST-2 ubiquitination levels were then detected by Western blot. (**C**) HEK293T cells were co-transfected with Myc-Ub, OTUD1, and FLAG-BST-2 or FLAG-BST-2-K18R, FLAG-BST-2-K21R, FLAG-BST-2-K47R, FLAG-BST-2-K79R, FLAG-BST-2-K106R, FLAG-BST-2-K111R, FLAG-BST-2-K112R, FLAG-BST-2-K126R, FLAG-BST-2-K151R, and FLAG-BST-2-K152R, followed by co-incubation with FLAG immunomagnetic beads after 48 h. BST-2 ubiquitination levels were then detected by immunoprecipitation. (**D**) HEK293T cells were co-transfected with Myc-OTUD1, HA-Ub, and FLAG-BST-2-K21R, followed by co-incubation with FLAG immunomagnetic beads after 48 h. BST-2 ubiquitination levels were then detected by Western blot. *n* = 3.

Different types of ubiquitin chains are known to drive distinct biological outcomes. To explore how OTUD1 modulates polyubiquitination of BST-2, the effects of OTUD1 on specific ubiquitin linkages were examined. We investigated OTUD1-regulated ubiquitination in HEK293T cells. The results showed that knockdown of OTUD1 significantly increased both K48- and K63-linked ubiquitination of BST-2 (Figure 5A). Conversely, overexpression of OTUD1 significantly reduced K48- and K63-linked ubiquitination of BST-2 (Figure 5B). BST-2 possesses 10 potential lysine residues that can serve as ubiquitination sites. To detect the specific lysine residue targeted by OTUD1, each of the 10 lysines were individually mutated to arginine. Interestingly, the mutation of lysine 21 completely abolished the reduction in BST-2 ubiquitination mediated by OTUD1 in HEK293T cells (Figure 5C), a finding replicated in HEK293T cells (Figure 5D). Collectively, these findings suggest that K21 is a critical site for the regulatory effects of OTUD1 on BST-2 ubiquitination, and that OTUD1 may promote BST-2 expression by specifically catalyzing the removal of K48- and K63-linked ubiquitin chains from K21 of BST-2. This is significant, given that K48-linked polyubiquitination is typically associated with protein degradation.

### 3.6. OTUD1 Potentiates the Inhibitory Effects of BST-2 on HIV-1 Release

**Figure 6:**
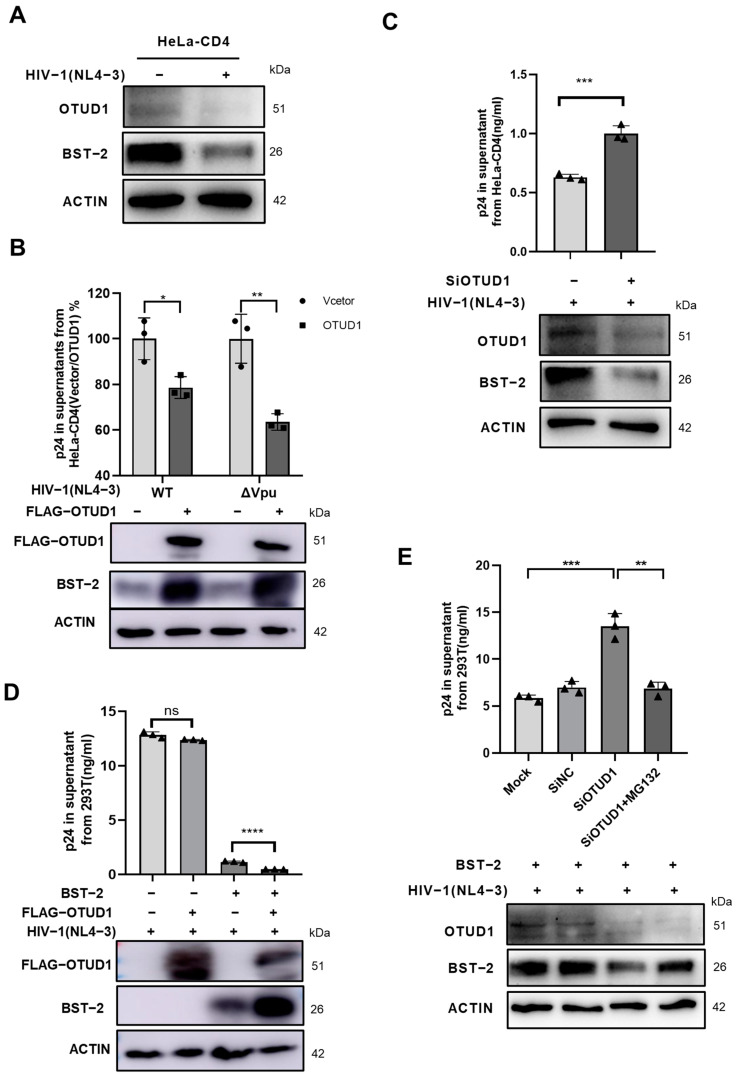
OTUD1 inhibits HIV-1 release by promoting BST-2 expression. (**A**) HeLa cells were transfected with HIV-1(NL4-3), with BST-2 protein levels then being detected by Western blot after 48 h. (**B**) HeLa cells were transfected with FLAG-OTUD1, HIV-1(NL4-3), or HIV-1(NL4-3) ΔVpu plasmids, with the supernatant and cells then being collected after 48 h. Expression levels of p24 and BST-2 were then detected by ELISA (YEASEN, Shanghai, China) and Western blot, respectively. (**C**) HeLa cells were transfected with OTUD1-specific siRNA, followed by supernatant and cell detection of p24 and BST-2 expression levels after 48 h by ELISA and Western blot, respectively. (**D**) HEK293T cells were co-transfected with FLAG-BST-2, FLAG-OTUD1, and HIV-1(NL4-3) for 48 h, followed by supernatant and cell detection of p24 and BST-2 expression levels by ELISA and Western blot, respectively. (**E**) HEK293T cells were co-transfected with FLAG-BST-2, OTUD1, and HIV-1(NL4-3). After 36 h, cells were treated with MG132 (10 μM) for 12 h, followed by supernatant and cell detection of p24 and BST-2 expression levels by ELISA and Western blot, respectively. *n* = 3. Bars and error bars indicate mean ± SD. Paired *t*-tests were used to compare groups. *n* = 3. * *p* < 0.05, ** *p* < 0.01, *** *p* < 0.001, **** *p* < 0.0001. ns, not significant.

The HIV-1 accessory protein Vpu is known to antagonize the host restriction factor BST-2 [5]. Notably, our results showed a significant decline in BST-2 levels in HeLa cells transfected with an infectious HIV-1 clone plasmid (pNL4-3) compared to mock-transfected cells (Figure 6A). Notably, we found that overexpressing OTUD1 led to a 21.39% decrease in HIV-1 p24 levels with HIV-1 Vpu and a 36.48% decrease with HIV-1ΔVpu (Figure 6B). P24 levels in culture supernatants were measured by ELISA (YEASEN, China). These results suggest that up-regulation of OTUD1 inhibits the release of HIV-1 virions by stabilizing BST-2. In addition, the results show that the effect of OTUD1 on HIV-1 replication appears to be more pronounced in the absence of Vpu. In contrast, down-regulation of OTUD1 led to an increase in the release of HIV-1 virions into the culture supernatant (Figure 6C), and OTUD1 inhibition of HIV-1 was also observed in Jurkat cells (Figure S3A,B). To further verify the effect of OTUD1 on the release of HIV-1, we transfected different concentrations of OTUD1 with or without BST-2-FLAG into Hela-CD4 cells, and then infected them with HIV-1 virus. We found that OTUD1 significantly inhibited the release of HIV-1 virus (Figure S3C). In addition, OTUD1 appears to have a synergistic promoting effect on the inhibition release of HIV-1 in the presence of BST-2-FLAG (Figure S3D). However, in HEK293T cells, which lack BST-2 expression, OTUD1 up-regulation had no effect on HIV-1 release (Figure 6D), whereas after overexpression of BST-2 in HEK293T cells, the amount of HIV-1 in the cell supernatant was significantly reduced, indicating that OTUD1 regulates HIV-1 virion release via the modulation of BST-2. Furthermore, administration of the proteasome inhibitor MG132 confirmed the inhibitory effects of OTUD1 on HIV-1 virion release via the stabilization of BST-2 protein levels (Figure 6E).

## 4. Discussion

As a critical protein degradation pathway, the UPS governs a wide range of cellular processes and plays an integral role in regulating various HIV-1 life cycle stages [22]. Research has shown that the UPS directly influences the expression of HIV-1 proteins, thereby promoting or inhibiting viral replication. For instance, the E3 ubiquitin ligase c-Cbl targets the HIV-1 Nef protein, leading to its ubiquitination and degradation via the K48 ubiquitin chain, which, in turn, inhibits HIV-1 replication [23]. In addition, the UPS can indirectly impact the HIV-1 life cycle by modulating host restriction factors. For example, the DUB USP49 affects HIV-1 replication by acting on the host restriction factor APOBEC3G [12]. In the present study, DUB OTUD1 was identified as a key regulator that stabilizes BST-2 protein levels and indirectly inhibits HIV-1 release via regulation of BST-2 ubiquitination. Thus, these findings reveal a novel antiviral mechanism wherein OTUD1 suppresses HIV-1 release by regulating host restriction factor BST-2.

OTUD1 has emerged as a critical regulator of various biological processes, including cancer progression, antiviral defense, and inflammatory responses [24]. Notably, OTUD1 has been implicated in modulating antifungal innate immunity through the deubiquitination of CARD9 [25], while its deficiency in various human cancers is strongly linked to cell survival, apoptosis [26,27], and the inhibition of cancer progression by triggering immunogenic cell death [28,29]. In this study, a novel OTUD1 function was discovered, whereby HIV-1 virion release was inhibited through the stabilization of BST-2 protein levels, revealing its previously unknown potential as an anti-HIV-1 factor.

Previous studies have demonstrated that OTUD1 preferentially hydrolyzes K63-linked ubiquitin chains in vitro [30]. However, OTUD1 has also been shown to regulate other types of ubiquitin chains, including K6, K27, K33, and K48 [31]. Notably, OTUD1 negatively regulates type I interferon induction by reducing K6-linked ubiquitination of IRF3 [32]. Moreover, OTUD1 reduces the K48 and K63-linked ubiquitination of Smurf1, enhancing Smurf1 expression and promoting the degradation of MAVS/TRAF3/TRAF6 signaling complexes, thereby down-regulating the innate immune response [33]. In our study, OTUD1 was found to reduce the K48- and K63-linked ubiquitination of BST-2.

BST-2 is a multifunctional protein involved in antiviral defense, cell signaling, and immune regulation, with inhibitory effects on malignancy [34,35,36]. The HIV-1 accessory protein Vpu is known to induce the degradation of BST-2 through ubiquitination by interacting with its transmembrane domain [37]. Our results showed that OTUD1 specifically reduced BST-2 ubiquitination at the lysine 21 residue, which is located in its cytosolic domain. Unfortunately, in this study, we did not directly explore the possible effect of BST-2-K21R mutation on HIV-1 virus release. Notably, in the absence of BST-2, OTUD1 had no effect on viral release, indicating that OTUD1 exerted its antiviral effects indirectly by regulating BST-2. Given that CD4+ T lymphocytes and macrophages are the primary target cells of HIV-1 [38], a shortcoming of this study is that experiments were not performed on primary cells, and future research should explore the impact of OTUD1 on HIV-1 release in these cell types.

Previous studies have suggested that Vpu-mediated ubiquitination of BST-2 does not rely on lysine, serine, or threonine residues within the cytoplasmic structural domain of BST-2, implying that Vpu may target other residues such as tyrosine or NH2-terminal methionine [39]. Additionally, the serine–threonine–serine sequence within the cytoplasmic domain of BST-2 has been identified as essential for Vpu-mediated down-regulation of BST-2 and viral release, emphasizing the importance of non-lysine residues, especially serine and threonine, in the ubiquitination process. These findings suggest that the interaction between Vpu and BST-2 may involve multiple mechanisms, including but not limited to ubiquitination pathways [40]. In addition, in this study, we only used labeled proteins to verify the interaction between OTUD1 and BST-2, and did not verify endogenous protein interaction or deeply explore the relationship between OTUD1, BST-2 and Vpu. Overall, our study characterized the relationship between OTUD1 and BST-2, providing clues to the anti-HIV-1 mechanism of BST-2.

## 5. Conclusions

In conclusion, this study identified OTUD1 as a novel deubiquitinating enzyme that inhibits HIV-1 virion release by reducing K48- and K63-linked ubiquitination of BST-2, thereby stabilizing its protein levels. This discovery highlights a previously unrecognized regulatory mechanism for HIV-1 host restriction factors and suggests potential avenues for anti-HIV-1 therapeutic development.

## Data Availability

The authors declare that all data supporting the findings of this study are available in the article and its Supplementary Information file.

## Supplementary Materials

The following supporting information can be downloaded at https://www.mdpi.com/article/10.3390/v17020260/s1: Figure S1: Screening of DUB-siRNA libraries that regulate BST-2 expression levels on membrane surfaces (The first round); Figure S2: Screening of DUB-siRNA libraries that regulate BST-2 expression levels on membrane surfaces (The second round); Figure S3: OTUD1 inhibits HIV-1 release by influencing BST-2 expression in Jurkat and Hela-CD4 cells; Table S1: Primers related to the gene synthesis; Table S2: Primers related to RT-qPCR.
