# Supplementary material for: The Deubiquitinase OTUD1 Influences HIV-1 Release by Regulating the Host Restriction Factor BST-2
Source: Viruses. 2025 Feb 14;17(2):260. doi: 10.3390/v17020260 (PMC11860778; doi:10.3390/v17020260)
Supplement: Supplementary file 1 [file viruses-17-00260-s001.zip › viruses-3380601-supplementary.pdf]

Supplementary information

The deubiquitinase OTUD1 influences HIV-1 release by regulating the host restriction factor BST-2

Man-Di Zhang [1,2,3, #], Fan Chen [2,3 #], Wen-Qiang He [1,4, #], Ying Lu [1], Feng-Liang Liu [1], Hong-Guang Zhang [3], Liu-Meng Yang [1], Chun-Sheng Dong [3, *], Si-Dong Xiong [2,3, *], Yong-Tang Zheng [1,2, *]

Supplemental Information contents:

Supplementary Figures S1-S3, Page 2 – Page 5.

Supplementary Tables S1-S2, Page 6-7.

Supplemental Figures

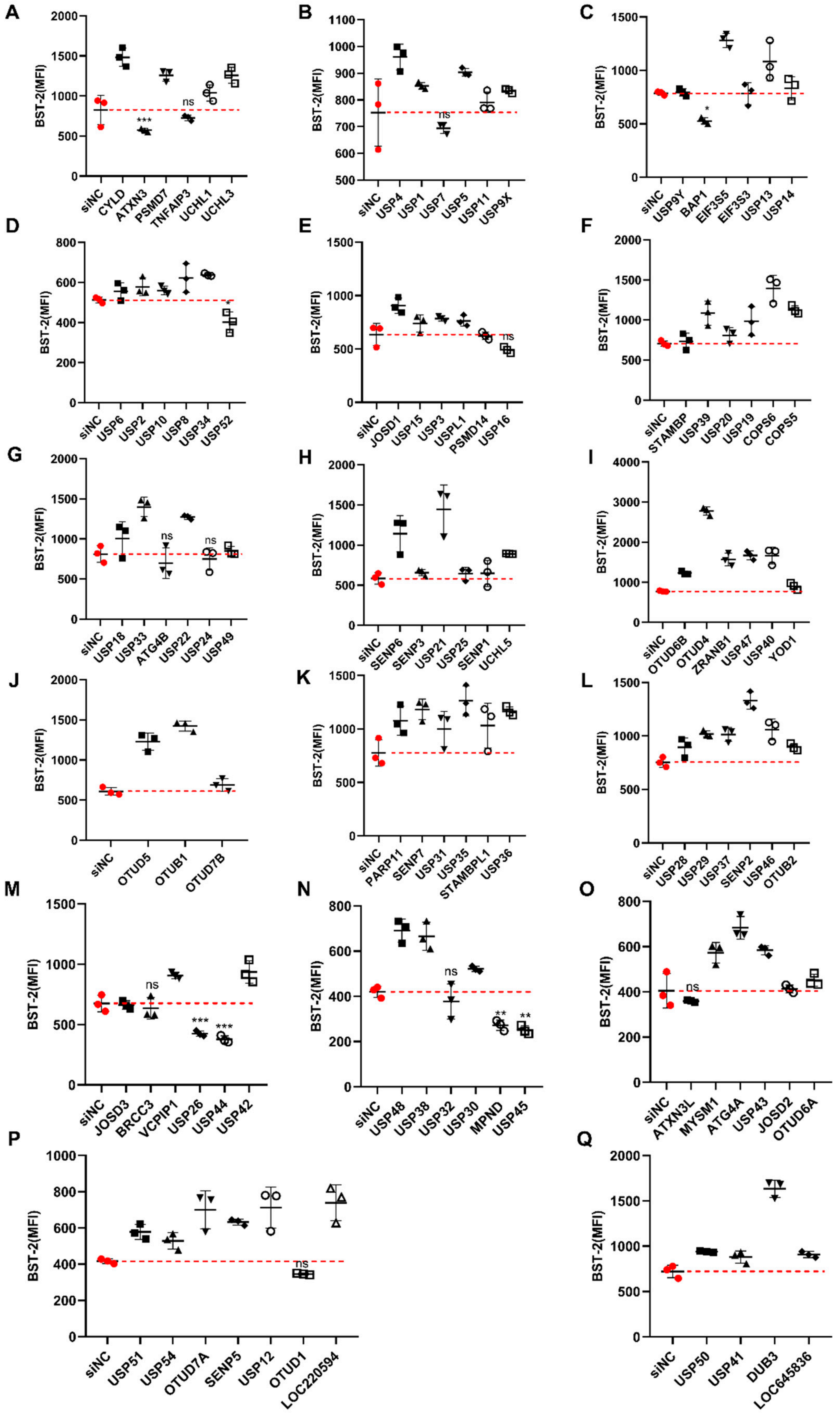

Figure S1. Screening of DUB-siRNA libraries that regulate BST-2 expression levels on membrane surfaces (The first round). HeLa cells were transfected with siNC and 98 DUB-siRNA for 48 h, then the surface BST-2 expression level was detected by flow cytometry. Data are presented as mean ± standard deviation (SD). (two-tailed paired t-test). *p < 0.05; **p < 0.01; ***p < 0.001; ****p < 0.0001.

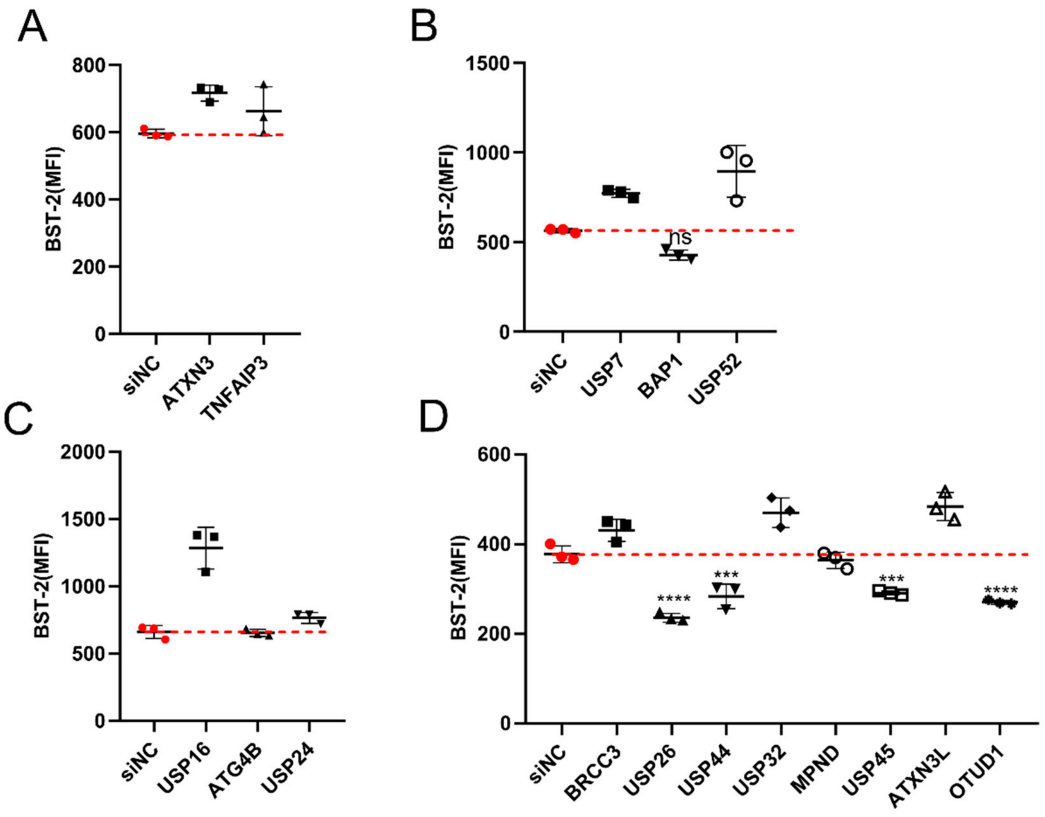

Figure S2. Screening of DUB-siRNA libraries that regulate BST-2 expression levels on membrane surfaces (The second round). HeLa cells were transfected with siNC and 16 DUB-siRNA for 48 h, then the surface BST-2 expression level was detected by flow cytometry. Data are presented as mean ± standard deviation (SD). (two-tailed paired t-test). *p < 0.05; **p < 0.01; ***p < 0.001; ****p < 0.0001.

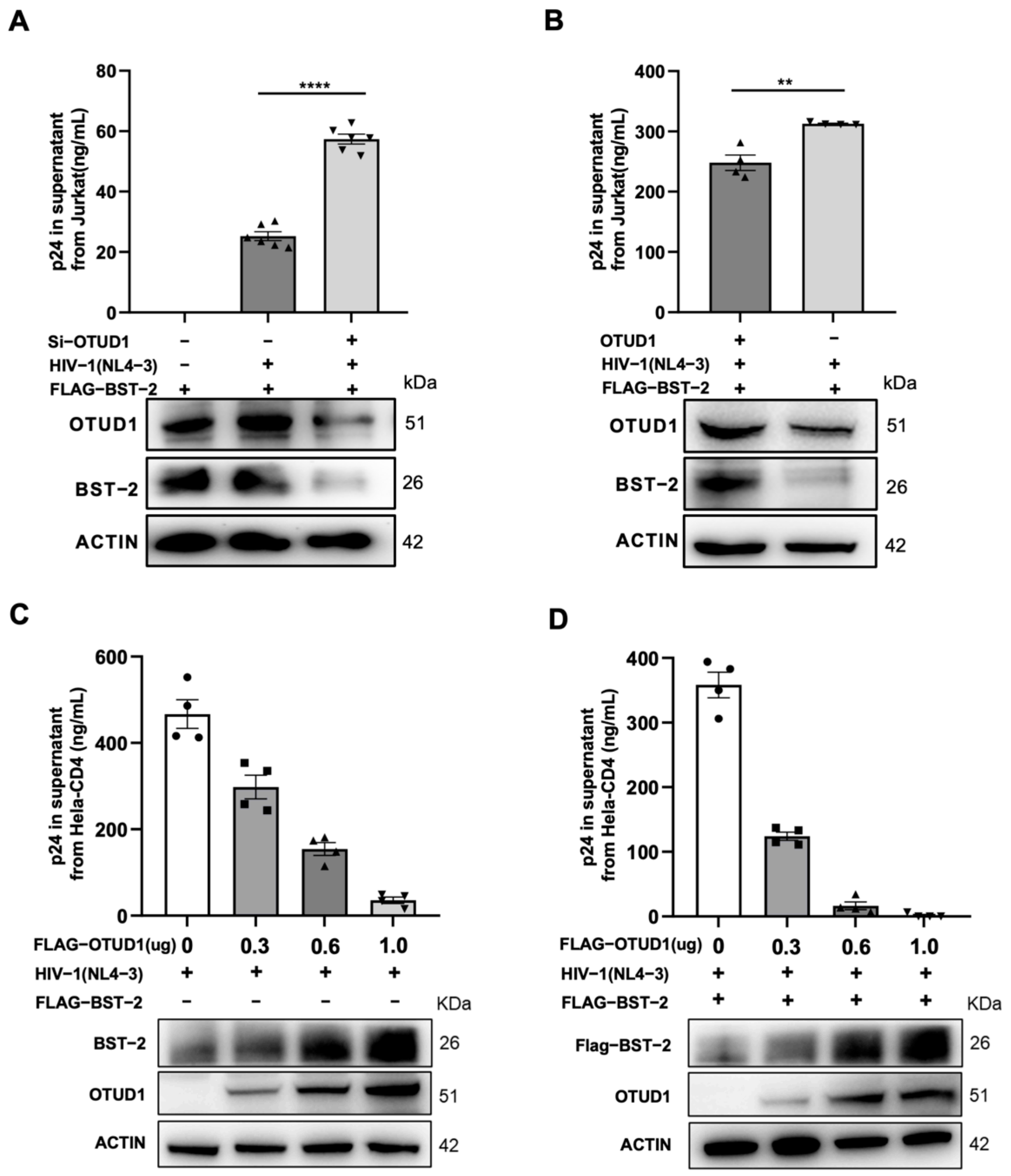

Figure S3. OTUD1 inhibits HIV-1 release by influencing BST-2 expression in Jurkat and Hela-CD4 cells. (A) Jurkat cells were transfected with OTUD1-specific siRNA, FLAG-BST-2 and pro-HIV-1 for 48 h, then the supernatant protein p24 was detected by ELISA, and the expression level of BST-2 in the cells was detected by western blotting. (B) Jurkat cells were transfected with OTUD1, FLAG-BST-2 and pro-HIV-1 for 48 h, then the supernatant protein p24 was detected by ELISA, and the expression level of BST-2 in the cells was detected by western blotting. (C-D) Hela-CD4 cells were transfected with different doses of OTUD1, with or without FLAG-

BST-2 for 24 h, then infected with HIV-1(NL4-3) for 48h, the supernatant protein p24 was detected by ELISA, and the expression level of BST-2 and OTUD1 in the cells was detected by western blotting. Data are presented as mean ± standard deviation (SD). (two-tailed paired t-test). $^{**}p < 0.01$; $^{****}p < 0.0001$.

Table S1. Primers related to the gene synthesis

Name	Reference Sequence	Primer Sequence (5′ - 3′)
**FLAG-OTUD1-F**	NM_001145373.3	AAGCTT ATGCAGCTCTACAGCAGCGTCT
**FLAG-OTUD1-R**		GAATTCTCAAGAGCATGCATTTTGTTCAATA
**FLAG-BST-2-F**	NM_004335.4	GAAGATCTATGGCATCTACTTCGTATGACTATT
**FLAG-BST-2-R**		CGGGATCCTCACTGCAGCAGAGCGCT
**OTUD1 C320S mutation-F**	NM_001145373.3	CTCGGTAGAGGCTGTTGCCGTCTGGA
**OTUD1 C320S mutation-R**		TCCAGACGGCAACAGCCTCTACCGAGC
**BST-2 K18R mutation-F**	NM_004335.4	CTTACAGCGCCTATCCCCGTCTTCCATGGG
**BST-2 K18R mutation-R**		CCCATGGAAGACGGGGATAGGCGCTGTAAG
**BST-2 K21 Rmutation-F**		CCCCAGCAGAAGCCTACAGCGCTTATCCCC
**BST-2 K21R mutation-R**		GGGGATAAGCGCTGTAGGCTTCTGCTGGGG
**BST-2 K47R mutation-F**		TCGCTGTTGGCCCTGATGGTGAAGATAATCAAGGGC
**BST-2 K47R mutation-R**		GCCCTTGATTATCTTCACCATCAGGGCCAACAGCGA
**BST-2 K79R mutation-F**		TGAAAGCCCCTCTGGGCCTCGGTCAGC
**BST-2 K79R mutation-R**		GCTGACCGAGGCCCAGAGGGGCTTTCA
**BST-2 K106R mutation-F**		TCCTTGGGCCCTCTCTGCATCCAGGGAAGC
**BST-2 K106R mutation-R**		GCTTCCCTGGATGCAGAGAGGGCCCAAGGA
**BST-2 K111R mutation-F**		CAAGCTCCTCCACTTTCCTTTGTCCTTGGGCCTTC
**BST-2K111R mutation-R**		GAAGGCCCAAGGACAAAGGAAAGTGGAGGAGCTG
**BST-2 K112R mutation-F**		GCTCCTCCACTCTCTTTTGTCCTTGGGCCTTCT
**BST-2 K112R mutation-R**		AGAAGGCCCAAGGACAAAAGAGAGTGGAGGAGC
**BST-2K126RCmutation-F**		GCGTCCTGAAGCCTATGGTTTAATGTAGTGATCTCTC
**BST-2 K126R mutation-R**		AGAGATCACTACATTAAACCATAGGCTTCAGGACG
**BST-2 K151R mutation-F**		GCTGGGGTAGTACTTCCTGTCCGCGATTCTCAC
**BST-2 K151R mutation-R**		GTGAGAATCGCGGACAGGAAGTACTACCCCAGC
**BST-2 K152R mutation-F**		GAGCTGGGGTAGTACCTCTTGTCCGCGATTC

**BST-2 K152R mutation-R**		GAATCGCGGACAAGAGGTACTACCCCAGCTC

Table S2. Primers related to RT-qPCR

**Name**	**Primer Sequence (5′ - 3′)**
OTUD1-Forward primer	TTTATCATCGCTGCTGCCCA
OTUD1-Reverse primer	GTCGCGTTTCCTTTGCACTT
BST-2-Forward primer	TTAAGCGTGAGAATCGCGGA
BST-2-Reverse primer	CTAACCGTGTTGCCCCATGA
GAPDH-Forward primer	TGCACCACCAACTGCTTAGC
GAPDH-Reverse primer	GGCATGGACTGTGGTCATGAG
